# Value-Based Caching in Information-Centric Wireless Body Area Networks

**DOI:** 10.3390/s17010181

**Published:** 2017-01-19

**Authors:** Fadi M. Al-Turjman, Muhammad Imran, Athanasios V. Vasilakos

**Affiliations:** 1 Department of Computer Engineering, Middle East Technical University, Northern Cyprus Campus, 99738 Kalkanli, Güzelyurt, Mersin 10, Turkey; fadi@metu.edu.tr; 2 College of Computer and Information Sciences, King Saud University, Riyadh 11543, Saudi Arabia; 3 Department of Computer Science, Electrical and Space Engineering, Lulea University of Technology, Luleå 97187, Sweden; athanasios.vasilakos@ltu.se

**Keywords:** caching, Information-Centric Networks, Wireless Body Area Networks, FIFO, LRU

## Abstract

We propose a resilient cache replacement approach based on a Value of sensed Information (VoI) policy. To resolve and fetch content when the origin is not available due to isolated in-network nodes (fragmentation) and harsh operational conditions, we exploit a content caching approach. Our approach depends on four functional parameters in sensory Wireless Body Area Networks (WBANs). These four parameters are: age of data based on periodic request, popularity of on-demand requests, communication interference cost, and the duration for which the sensor node is required to operate in active mode to capture the sensed readings. These parameters are considered together to assign a value to the cached data to retain the most valuable information in the cache for prolonged time periods. The higher the value, the longer the duration for which the data will be retained in the cache. This caching strategy provides significant availability for most valuable and difficult to retrieve data in the WBANs. Extensive simulations are performed to compare the proposed scheme against other significant caching schemes in the literature while varying critical aspects in WBANs (e.g., data popularity, cache size, publisher load, connectivity-degree, and severe probabilities of node failures). These simulation results indicate that the proposed VoI-based approach is a valid tool for the retrieval of cached content in disruptive and challenging scenarios, such as the one experienced in WBANs, since it allows the retrieval of content for a long period even while experiencing severe in-network node failures.

## 1. Introduction

Great interest is currently invested in Wireless Body Area Networks (WBANs) used for remotely monitoring and reporting users’ health vitals [1]. Composed of various sensors, both wearable and implanted, a WBAN relies on wireless connectivity to transfer the collected data to an Internet-based data management service [2,3]. Applications span healthcare, sports, and industry, and have received attention from various commercial and civilian sectors. A healthcare example is that of remotely monitoring a patient’s condition, allowing patients to maintain lifestyles and comfort while offsetting hospital management costs. Such a setup would facilitate giving early warnings to patients with heart or neurological problems, and providing a “heads up” for nearby caregivers. Similar systems can be applied for airplane pilots and long route drivers to alert them to their awareness level. WBANs can also be fitted to workers in harsh environments, such as mines, to avert serious consequences from physical or chemical strain. A typical system comprises the following components: (1) a fitted WBAN; (2) a gateway between the WBAN and the Internet; (3) a service for data transfer over the Internet; and (4) a service for data processing, providing interface and alerts to the caregivers. For a successful and reliable WBAN operation, a harmonious nearby set of data resources need to be simultaneously engaged in all components. The human body is a challenging terrain for wireless communication, especially given the WBAN power constraints (due to limitations on the amount of energy allowed to penetrate the human body). Accommodating various levels of harshness in the surroundings in terms of temperature, dust, humidity, etc., dictates a resilience requirement to an expanded set of failure possibilities that includes partial or complete failure of the WBAN nodes and reduced levels of activity or accuracy as batteries deplete, which form serious threats for losing critical in-network data before it is utilized. Equally, a WBAN needs to maintain high integrity for the data collected. This requirement stresses both the integrity of *in-network storage* elements and the computations of the collected data at the different components. Moreover, a fitted WBAN needs to sustain different levels of mobility. Since the WBAN elements rely on each other to gather and process data, mobility may be temporarily or permanently detrimental to the network operation by breaking some functional communication links that affect the in-network data retrieval.

Hence, nodes and links are prone to several risks leading to high probabilities of failures, and several nodes in the WBAN may become disconnected. We are characterizing such circumstances by the Probability of Node Failure (PNF) and Probability of Link Failure (PLF). At the same time, network elements rely on instantaneous data updates in order to maintain healthy user conditions. Delivery and accessibility of in-network data can, hence, be extremely challenging, especially when dealing with highly variable data such as neurological signals which necessitate timely recording. Thus, delay/latency in accessing data forms a critical design factor in these networks. Meanwhile, the priority of different collected data depends on the nature of the monitored phenomena and the criticality of the user’s condition. Therefore, a prioritization that can be either preset or reactive to the user’s condition at both the WBAN level and during transfer to the caregivers is needed. Consequently, for a successful and reliable operation and monitoring of the WBAN system, ther Information Centric Network (ICN) paradigm shall be applied in such systems for immediate in-network data access.

ICN is the next generation model for the Internet that can cope with the user’s requests/inquiries regardless of their data-hosts’ locations and/or nature [4]. The current Internet model is suffering from the exchange of huge amounts of data while still relying on the very basic network resources and IP-based protocols. Meanwhile, ICNs promise to overcome major communication issues related to the massive amounts of distributed data in the Internet. ICNs adopt a content-centric architecture which focuses more on the networked data itself rather than the meta-data. Luckily, the architecture of these ICNs closely match with the emerging communication trend that aims at exchanging Big-data over tiny and energy-limited WBAN in order to realize numerous attractive projects such as the Smart-planet and the Internet of Things [5,6,7].

In order to enable WBAN to support this trend in communication and function as a reliable platform, we proposed the cognitive framework in our previous work [8]. In [8], an information-centric scheme is proposed for typical Wireless Sensor Networks (WSNs) using *cognitive* (intelligent) in-network devices that make dynamic routing decisions based on specific *Knowledge-* and *Reasoning-*observations in the WSNs. *Knowledge* representation using the <*attribute*, *value*> pair, and *Reasoning* using an analytic hierarchy process (AHP) techniques are employed at the cognitive device in order to decide on the best data route. AHP can be applied on quality of information (QoI) attributes in next generation WBANs such as reliability, delay, and network throughput observed over the communication links/paths [9,10]. The QoI is defined in this work as the level of satisfaction experienced/perceived by the end-user about the received information from the WBAN. Attributes such as reliability, latency, and throughput are used to evaluate the QoI. It is worth pointing out here that QoI is not referring to the Quality of Service (QoS) term used in typical WSNs [3]. In fact, QoS takes care of the operational aspects of the network, while QoI is associated with the characteristics of the sensory information made available to the end-user/sink-node.

Cognitive WBAN is able to significantly outperform the *non-cognitive* WBAN paradigms [11]. However, this cognitive WBAN framework necessitates a reliable in-network caching feature. In this paper, we propose the use of a Value of sensed Information (VoI) cache replacement strategy. It identifies the most suitable data to be replaced in order to maintain prolonged data availability periods. Where the VoI is defined as the associated value with a piece of information in the WBAN that accumulates the data age, popularity, delay, and energy depletion cost factors based on adjustable tuning parameters.

Unluckily, traditional cache replacement strategies in the literature [12,13] have been designed mainly for IP-based computer networks and data-centers, which have distinct characteristics in locating data from the envisioned next generation networks such as the light-weight WBANs. In fact, choosing the most appropriate caching strategy can have significant implications on the overall network performance in terms of the data *publisher’s load*, *hit-ratio* and *time-to-hit* metrics. Several works in the literature have addressed each of these metrics separately. However, since a single WBAN can serve numerous kinds of applications/users with varying design requirements, we believe in the necessity of a generic dynamic utility function that can consider all the aforementioned metrics while setting different weights for each depending on the WBAN application.

To this end, we provide a novel utility function that sets a value to each cached data item in a cognitive WBAN framework. We provide a cache replacement strategy that depends on the VoI in choosing the most appropriate data to be replaced in the cache. We compare our VoI-based strategy against two other significant approaches in the literature, that we call Least Recently Used (LRU), First In First Out (FIFO), and Least Value First (LVF), while considering varying parameters in ICN-WBAN including the *cache size*, and *requests’ counts*. Moreover, we testify our caching approach under flat and hierarchical storage structures. In the flat structure, we assume only one level of caching. However, in the hierarchical one, we assume two levels of caching.

The rest of the sections in this paper are organized as follows: A literature review on caching approaches in ICN-based WBANs is provided in Section 2. In Section 3, we introduce our WBAN-specific system model based on which we build our proposed caching strategy. Section 4 explains the proposed VoI approach, utility function, and theoretical analysis. Section 5 presents extensive simulation results of the VoI in comparison to other caching strategies. And finally, we conclude our work in Section 6.

## 2. Related Work

WBANs can be viewed as a special type of content-oriented Wireless Sensor Network (WSN), and, as such, they inherit some of a WSNs’ general characteristics. The uniqueness of WBANs as a paradigm, however, mainly stems from dealing with the terrain of the human body. The human body, as a communication terrain, poses unusual challenges for both in-body and in-air communication. Many factors including body mobility, changes in posture, size, weight, or water content, in addition to co-existence of different technologies in the same band, affect the throughput of the WBAN. For life threatening cases, for instance, the resulting data packet loss/miss is simply unaffordable. Moreover, essential considerations for safety and comfort prohibit the use of capable transmission, sensing, and processing techniques [1,2]. A substantial component, as well, of the WBAN operation involves processing some measured information at the network level, i.e., before delivering it to the gateway. Leading motivators for in-network caching include energy savings, reduced delay, measurement aggregation (summary, average, etc.), measurement verification, etc. [14]. In general, given the nature of the WBAN application, there is a high demand for operational reliability in terms of data availability and accessibility. While such considerations do not dismiss the plethora of work with WSNs in the literature, certain challenges that are unique to WBANs call for novel solutions. Within the context of WBANs, there is an apparent void in addressing the challenges in data caching, which has always been treated as a key feature in ICNs. In this paper, we propose the use of an ICN-based solutions in addressing this void in WBANs in order to augment and provide more in-network data-hits in a timely manner.

At the core of ICNs, data have to “live” near their requesters. This is the task of caching schemes. Thus, efficient caching mandates two properties: (1) ensuring an updated copy of the requested data resides at entities close to the region of interest (in terms of reachability latency); (2) that copy remains “live” for as long as interest in it exists. Caching is coupled with routing and naming architectures. For example, in Data-Oriented Network Architecture (DONA), the coupling of naming tuples—Owner public key with data Label—enables in-network caching for any entity in the network that could hold a valid copy. Architectures differ in deciding which entity is allowed a copy of the data, and the basis upon which each entity would retain the data. A recent effort in age based caching argues for a two-fold metric for caching a NDO replica. If it resides at a network edge, or has higher popularity, it will remain cached for a longer duration. Entities which hold replicas will collaborate in “tuning” the age counter to manifest such factors. A core disadvantage at many caching protocols is the inherent need for book-keeping. The resulting message-exchange overhead cannot scale to the Internet and remain efficient. We are bound to analyze caching schemes under the following conditions: (i) Communication overhead per NDO; (ii) Storage requirements; (iii) NDOs with different priorities; and (iv) Granularity in assessing request frequency, types, locations, etc. We review the different caching techniques in ICNs and identify the techniques that are best suited for the caching decisions in WBANs. The in-network caching in ICN-based WBANs can be categorized into the following categories: (*A) Location-based caching; (B) Content-based caching; and (C) Functionality-based caching.*

### 2.1. Location Based Caching

Chai et al. in [15] have argued against caching the data everywhere in ICNs and recommended caching less in order to achieve better network performance. Their caching policy claims that data shall be only cached at the nodes having the highest probability of getting a cache-hit on the data delivery path. Eum et al. [16] have proposed a Content-Oriented Network (CON) architecture, called Cache Aware Target idenTification (CATT). This architecture assumes a topology-aware caching policy, where a node on a downloading path is selected for caching as long as it has the highest connectivity-degree based on the geographical location of the node. However, this kind of node can form a geographical bottleneck in the network. Meanwhile, authors in [17] have investigated the performance of topology-based replica placement on Internet router-level topology and found that the router-level fan-out placement is almost as good as that of the greedy placement of the replica. Moreover, they found that a fan-out based replica placement method needs to be carefully designed to be efficient in content-oriented architectures.

Works in [15,16,17] are based on the node degree or node fan-out for replica placement, but these methods cannot be universal because node degree-based solutions cannot be good solutions if most of the nodes have a similar, relatively low degree or fan-out. Bhattacharjee et al. [18] have considered the use of various self-organizing or active cache management strategies in which nodes make globally consistent decisions about caching and revealed that in many cases, these self-organizing caching schemes yield better average delays than traditional approaches (Cache at transit nodes), using much smaller per-node caches. A Selective Neighbor Caching approach which selects an appropriate subset of neighboring proxies that minimize the mobility costs in terms of expected average delay and caching costs has been proposed in [19]. This approach is based on proactively caching data requests and the corresponding meta-data to a subset of proxies that are one hop away from the proxy. This kind of caching is usually following a FIFO technique in replacing the cached data based on its in-network locality.

### 2.2. Content Based Caching

The main objective of the work in [20] is to minimize the Internet service provider (ISP) traffic and accessed in-network devices by caching frequently requested data at ISP-specific routers. The main problem addressed here is to provide effective caching strategies for these routers to coordinate their data replacement based on their content. Guided by optimal replica placement, the authors have presented two popularity-based caching algorithms. However, this work may not be practical as authors have assumed only one gateway in an ISP network.

Cho et al. [21] have proposed a content caching approach, called WAVE, in which the cache size is adjusted based on data popularity. In WAVE, an upstream node recommends the number of chunks to be cached at its downstream node, which increases exponentially as the data requests increases in order to reduce communication and cache management overhead. WAVE distributes content chunks towards the network edge (from where the data requests come) considering the content popularity and distance relation. However, the different sizes of data chunks have not been considered in this reference. An age-based distributed cache approach aiming at reducing the data publisher load and in-network delay for ICNs has been proposed in [22]. This approach provides a lightweight cooperative mechanism to control where data contents’ ages are dynamically updated implicitly. It spreads popular contents towards the edge of the ICN and, meanwhile, eliminates the unnecessary replicas at the intermediate ICN nodes. Yet this approach suffers from maintaining highly dynamic contents, and, thus, nodes which are far away from the server may experience long time periods to refresh their contents. This caching category can be represented by the famous term, Least Recently Used (LRU); as it replaces the outdated data based on the content itself rather than its locality.

### 2.3. Node Functionality Based Caching

To unleash the full potential of ICNs, the role (function) of the in-network caching node shall be taken into consideration and will consider which content to cache at the management/control level rather than guessing it at the data level. Authors in [23] remarked the side-effects of delegating the caching decision to the data level and propose a specific approach to handle data caching at the control level. The proposed approach can be tuned to create a balance between the benefits and costs overhead. However, it is only applicable at a small scale, and it may not accommodate the massive amounts of data contents in the Internet. In [24], authors investigated the trade-off between caching data contents in a distributed IP-based network and the new emerging ICN architectures such as the Content-Centric Network (CCN). They applied their study on the real traffic mix resulting from several functional resources such as the web, file sharing, and multimedia streaming. It has been demonstrated that caching videos in routers offers more cache hits. Nevertheless, the other types of content would likely be more efficiently handled in very large capacity storage devices in the core of the network. Thus, this kind of caching is not efficient in ICNs, and it follows the FIFO techniques in caching.

In this paper, although we need to use a content-centric approach, the traditional cache replacement approaches cannot be applied to an ICN-based WBAN. This is because of the unique resource constraints of the sensor network, the uncertainty of the wireless medium, and the need to be aware of application-specific requirements in ICN architectures. The resource limitations of the sensor nodes include limited power supply, storage space, and heterogeneity in terms of the sensors used and the node functions. In addition, the same content (sensed data) cannot be replicated into multiple caches without associating them with location, because the sensed information may be different in different parts of the network, and it may change over time too, which is unlike the case of ICNs. This makes cache replacement trickier in information-centric sensor networks. In addition, the replacement policy should take into account the type of user/application requests coming to the network, the sensor node availability at different locations (as nodes eventually die out), and also the sensing duration for different sensors on board the sensor nodes.

Consequently, the Internet has progressed towards more information centric paradigms, where the focus is on delivering named blocks of data to users at the network edge rather than establishing end-to-end connections to the web server. So the design of the cache replacement policy in ICNs must be a dynamic one, based on the user’s request trends and the application on hand. In [25], we proposed the concept of dynamic caching via assigning dynamic values for the exchanged in-network contents. Then, we chose the least valued ones to be replaced. Accordingly, this technique is called the Least Value First (LVF) approach. However, in LVF there was no cognition in making the caching decisions, and it assumed the traditional flat storage structure (i.e., one level of caching). In this paper, and unlike other related work [26,27], dynamic caching decisions are made based on specific Knowledge- and Reasoning- observations in the network. Based on these cognitive elements/observations, we prioritize between the cached contents in a hierarchical storage system (i.e., in a multi-level caching). Accordingly, we provide a novel utility function that sets a value to each data item based on application-specific metrics, such as required quality of communication channel, delay, and data age. This makes our proposed VoI approach able to cope with the next generation sensor networks trend in communication.

## 3. System Models

In this part of our work, we elaborate on our ICN-based WBAN model in addition to its corresponding data popularity, age, and delay models.

### 3.1. ICN-Based WBAN Model

**Figure 1:**
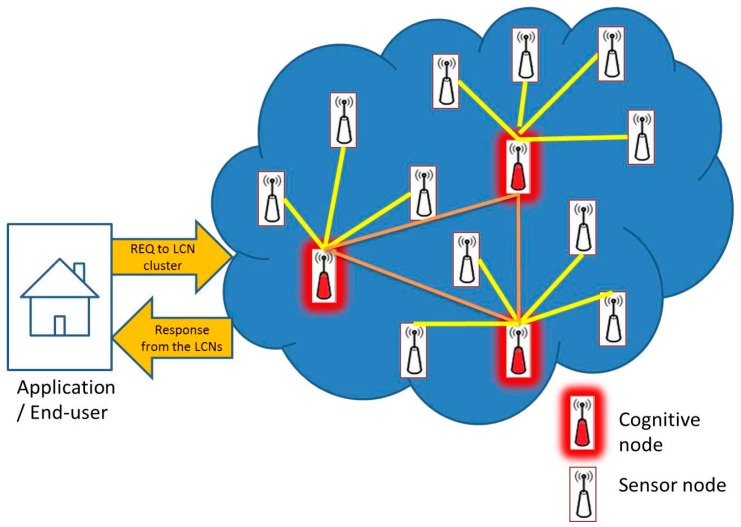
Network model with sensor nodes and cognitive nodes.

The main components in our ICN network model are listed as follows: Sensor Nodes (SNs) to sense the environment and capture physical changes in the surrounding environment, and report these changes via relay nodes (RNs) or local cognitive nodes (LCNs). They interact with the RNs and LCNs as shown in Figure 1. LCNs have elements of cognition, e.g., knowledge, reasoning, and learning, which help in interpreting common requests and queries’ responses. They interact with SNs, RNs, and the Sink. RNs forward the data received from SNs to the Sink or any neighboring local cognitive nodes in response to received requests from the user. The sink is where all the collected data is delivered. The sink node is also enhanced with cognitive elements to be more intelligent in managing the network performance based on data traffic type and it is called a Global Cognitive Node (GCN).

The type of data traffic that an ICN-based WBAN paradigm can handle is categorized into one of following: (1) *Type I: On-Demand*; (2) *Type II: Periodic*; and (3) *Type III: Emergency*. Each of these types is associated with a different Quality of Information (QoI) value on the cached data, based on the WBAN application. We select the network *Reliability (R)*, *Latency (L)*, *Energy (E)*, and *Throughput (T)* as the four main attributes, whose combined value decides the QoI of the cached data. We are not using the absolute values for these attributes. Instead, we associate priorities with each of these attributes for every request type, and make these priorities decide the importance of the absolute value of the attributes, as shown in Table 1. For instance, number 1 indicates the highest priority and number 3 indicates the lowest. The ‘x’ in Table 1 indicates a “do not care” condition. This means that there are no strict requirements on the value of the QoI marked with an ‘x’, and its value does not affect the caching decision.

### 3.2. Delay Model

Different sensors have different durations for which they need to be exposed to the environment so that they can capture the sensed readings accurately. This affects the duration of the on-time of the sensor node, which in turn affects the lifetime of the sensor node [28]. In order to prolong the lifetime of the sensor node, it is useful to store the sensed data for longer when the delay involved in acquiring the reading is longer. This is called the sensing delay. In addition, if data has to be propagated from sensor nodes to LCNs every time data is requested, it would add to the propagation delay of the data, especially if the sensor nodes are located far away from the Sink. Thus, the delay components we consider are the sensing delay δ and the propagation delay τ. Accordingly,

(1) τ∝n,

(2) $$\delta \propto \max(d_{1},d_{2},d_{3},..d_{k})$$

where *k* is the total number of sensors available on board of the sensor node, and *d_i_* represents the fixed sensing delay value of the sensor type *i* (see Table 1). Thus, the sensing delay is a function of the maximum delay from among the sensor types that have been activated to provide fresh data. Putting these two delays together, the total delay (∆) involved in delivering freshly sensed data to the sink is a combination of the sensing and propagation delay, given by Equation (3).

(3) Δ=τ+δ

### 3.3. Age Model

Our age model makes use of the following two conditions to decide what content should be dropped from the cache. The first is based on the periodicity of the periodic request (Type1 traffic), and the second is based on when the node’s cache is full. We make use of the periodicity of the periodic request, because freshly sensed data has to be provided at the start of each periodic request cycle. Thus, when the cache is full at the end of one periodic request cycle, old data can be discarded from the cache. Thus, the age of a sensed attribute-value pair is represented by its time-to-live (TTL) which is based on the periodicity of the request of each application type. This value is provided to the LCN by the GCN/Sink. Since we are not considering the use of historic data, our model implies that cached contents may be refreshed after every periodic time interval, as long as the data is being transmitted to the sink at the end of each cycle.

(4) $$TTL_{Si}\propto T_{periodic}$$

Equation (4) represents that the TTL of the sensed information (*Si*), represented as an attribute-value pair, is directly dependent on the periodicity of a request in Type 1 traffic flow. In case the application requires that the periodic data be stored for a prolonged duration of time, for example 24 h, before making a single transmission to the sink, the cache retention period becomes a function of the transmission cycle’s periodicity.

### 3.4. Popularity of On-Demand Requests

Traffic flow generated in response to on-demand requests have been classified as Type 2 traffic. More users may be interested in a particular type of sensed data, or a specific sensed data may be requested more times by one or more users. Such sensor data is said to be popular, and can be retained for longer in the LCN’s cache. Thus, the popularity of the sensed attribute-value pair is given by Equation (5).

(5) PopularitySi∝ReqSi/Reqtotal

where ReqSi is the total count of requests for an attribute-value pair received at an LCN, and Reqtotal is the total count of requests received by that LCN within a particular round of a ICN-based WBAN operation. In addition, when sensor nodes start to die out in the network, LCNs should store the data for longer to maintain their availability. When the primary LCN storing such data itself starts to die out, storing the data in neighboring LCNs provides extra storage guarantees and ensure availability of data in the network for longer. This storage requirement based on non-availability of alive sensor nodes is managed by the planning algorithm for data delivery based on the traffic flow in the network and remaining energy at LCNs.

### 3.5. Channel Communication Model

Here we elaborate on the assumed channel model in our wireless communications [29]. The transmission power utilized by the ICN-based WBAN nodes is represented as $T_{po}$ and the transmission range between the BS and an SN is represented as $T_{r}$. The expression of the channel model can be provided as:

(6) $$C_{M}=A\rho T_{P_{0}}T_{r}^{-\alpha }$$

where $C_{M}$ is the transmission power of the BS, A is the constant gain factor for power provided by the antenna, and amplifier gain, ρ, is the small scale constant for fading factor, and α is the path loss exponent. The transmission range between the WBAN nodes of i and j is denoted as $R_{ij}\;\left(i,j=1,2,3,\ldots \ldots ,N\right)$. The range for WBAN nodes (i and j) and the BS is denoted as $r_{i}$ and the power transmission for node i is defined as $p_{i}$. The link interference is expressed as:

(7) $$I_{BS}=\frac{A\rho T_{P_{0}}T_{r}^{-\alpha }}{A\rho T_{P_{i}}r_{i}^{-\alpha }}$$

## 4. VoI Cache Replacement

For cache replacement in ICNs, we need to ensure that we choose data appropriately for storage based on the following criteria: Firstly, data that takes longer to sense should be stored for longer to conserve the sensor node’s energy. Secondly, data storage must be a function of the periodicity of the requests based on the traffic type. This will help to store data until fresher data is available and to service requests for different traffic types in a timely manner. Lastly, value of the data based on its age, i.e., if temperature in a region has changed considerably from the last time it was sensed, then the cached information is stale and does not provide correct information. Hence, the freshness of data is also an important criterion when servicing requests for data on demand. Since these criteria are known and fixed, the cache replacement plan can be programmed into the LCN.

We propose a VoI-based cache replacement strategy for the LCNs in an ICN. Our cache replacement approach adopts the aforementioned system models to achieve an efficient cache management strategy that can handle the following three types of content:

1. Delay-based content: The delay sensitivity of the cached data content is a measure specified by the requesting user to indicate how long the consumer is willing to wait for it. Examples of delay-sensitive data can be found in applications serving areas of emergencies (e.g., disaster or health emergency).
2. Age-based content: Some contents are more sensitive to aging. For instance, if a user requests information about the traffic updates for the coming 30 min, then any related content that does not cover this time interval is useless.
3. Demand-based content: This is a measure of the data popularity which is specified by the frequency of requesting specific data.

Accordingly, the VoI-cache management approach employs three parameters to set a *value* $VoI_{Si}$ per sensor node *S_i_* reading. This value is dependent on the history of the data content within each operational round. At the beginning of each round and based on the aforementioned models, every content resets its $VoI_{Si}$ value according to the following function:

(8) $$VoI_{Si}=\alpha \times \Delta +\beta \times TTL_{Si}+\gamma \times Popularity_{Si}+\lambda \times 1/I_{BS}$$

where *α*, *β*, *γ* and *λ* are the tuning parameters that are specified based on the traffic type and the user requests. The strength of the VoI is mainly in its ability to prioritize based on the targeted WBAN application. Given the importance of the data popularity factor, especially in disaster cases, we would like to remark that it is recommended not to separate this factor totally from the proposed VoI concept. However, it is complementary with other important factors such as the age, delay, and cost in terms of the consumed energy and caused interference within the WBAN. Thus, to improve the basic priority caching method, the weights of each of the parameters can be adjusted to find the most efficient approach. The delay sensitivity parameter serves in assuring the least delay. The popularity parameter is important as it takes into consideration the most frequently demanded data packets. The packet age value is also vital since it considers packets in the cache that have not been used for a long time and replaces them with more relevant data. In the following, Algorithm 1 provides the steps to be executed by each node if its cache is full to drop data with the least $VoI_{Si}$.
**Algorithm 1:** Drop least $VoI_{Si}$.1. **Function VoI** (*content*)2. **Input**3. *content: A content item within the ICN.*4. **Begin**5. **for** each node, **do**6.  **for** each duty round, **do**7.  **Set**
*value* of each $VoI_{Si}$ in the cache based on Equation (8)8.  **if**
*cache_full*9.   Check history of the data requests10.    Drop the data content of the least $VoI_{Si}$11.   **End if**12.  **End for**13. **End for**14. **End**

### Theoretical Delay Analysis

One of the key objectives in VoI is to minimize the worst delay experienced between any sensor-node pair. Thus, we adopt the delay model introduced in [28]. Most importantly, we assume its discretized delay metric that can be tuned to achieve any desired accuracy. Due to dense network topologies formulated in WBAN, a relatively long multi-hop path can easily exist between the source node and the corresponding destination. Consequently, the delay components we are considering in this case are the transmission/processing delay 𝜓 represented by the number of hops multiplied by 𝜓, and the propagation delay, modeled based on the speed of signal and the Euclidian distance between the two ends (source and destination). The latter delay is extremely dependent on the speed of the link (or signal speed) and the Euclidian distance between the source and destination. It varies based on the utilized technology and its corresponding standards and the transmission medium.

Accordingly, the experienced delay in WBAN can be described as follows: We define a delay step ω which is the distance a wireless signal would travel in one time-unit. Assuming *E_ij_* is the Euclidian distance between a source node *i* and a destination node *j*, then the discrete propagation delay over a single-hop link (*i*, *j*) would be $\left\lceil \frac{E_{ij}}{\omega }\right\rceil$. Hence, the discrete delay over a multi-hop path is the sum of the discrete delays of single-hop links that constitute that path. Note that ω (and the time unit) can be made small enough to meet any desired accuracy. Thus, the upper-bound delay for a single-hop (*D_single_*) and multi-hop (*D_total_*), can be respectively defined as follows:

(9) $$D_{single}=\lceil \frac{E_{ij}}{\omega }+\psi \rceil$$

and

(10) $$D_{total}=\sum_{total\;hops}\lceil \frac{E_{ij}}{\omega }+\psi \rceil$$

## 5. Performance Evaluation

In this section, we provide initial performance evaluation results for the VoI-based cache replacement technique, which we have compared with FIFO, LRU, and LVF techniques using NS3 (https://www.nsnam.org/overview/media-kit/), a discrete event simulator. We adopt the aforementioned delay, popularity, and data aging models proposed in Section 3, which are described in Equations (3), (5) and (7). In our simulation, *r* is set to 142 mm, *P* is set to 3 dBm, data rate is set to 25 kbps. The packet size is 512 bits. Every WBAN node has an initial energy of 50 J and generates 5 pkts/s. Our simulations involve networks with 40, 60, 80, 100, 120 or 140 nodes randomly deployed in a field of radius R that range from 400 mm to 1400 mm. Thus, mesh topologies are formulated and assumed in this simulation. For each network size, we test 20 instances and take the average. To generate a trajectory for the requested data, we use the Directed Diffusion (DD) approach. As for the arbitrary variables specified in our VoI caching function in Equation (8), we set *α* = 0.2, *β* = 0.2, *γ* = 0.3, and *λ* = 0.3.

We make use of the Cache Hit Ratio to compare the performance of the different cache replacement strategies. Cache hit ratio is defined as the ratio of the number of times requested data was found in the cache divided by the total number of times data was requested from the cache. The storage cache is implemented as a single storage level in one case (L1 cache) and as a hierarchy of two storage levels in another case (L1 and L2 caches). Simulation results are compared for VoI, LVF, LRU, and FIFO replacement techniques. These simulations were run at cache sizes ranging from 10 to 100 Mbyte, and the simulations end after serving 1000 packet requests. There are 100 different requests from which the packet requests are randomly generated.

### 5.1. Performance Metrics

To compare the performance of the proposed VoI approach, we track ICN-specific metrics to achieve qualitative conclusions for the targeted in-network caching problem. We simulate the performance of an ICN-based WBAN with the detailed physical layer NS3 built-in parameters so that we achieve realistic simulation instances. The four considered performance metrics are as follows:

- Cache-hit ratio: is simply the fraction of time a request arrives at a node to which that cache is attached but does not contain the requested data item. It is the average hitting ratio over all the in-network caches. We preferred to look at average time to hit data and hitting ratio more than publisher load, but we generally expect publisher load to improve as the other metrics improve as well.
- Time-To-Hit-data (TTH): is found by simply logging all the total costs of the request and response paths incurred by every sensor node. Ideally, ICN-based WBAN is supposed to minimize the total average time-to-hit data per request
- In-network latency (delay): this metric represents the end-to-end delay as described above. Note that we differentiate between latency to hit data and in-network latency since the two metrics may differ because of mobility or disruption conditions
- Average Request per Publisher (ARP): this metric is measured in number of data requests per hour (req/h) and it represents the average load per publisher in an ICN paradigm. We track publisher load by monitoring the total fraction of data requests that had to be satisfied by a data publisher.

### 5.2. Simulation Parameters

Many of the ICN paradigm parameters have to remain fixed while our simulation instances are generated. In particular, the parameters of our simulation are as follow:

- Percentage of nodes with caches (PoC): This parameter is our primary method for controlling the extent of caching in our ICN. By varying this parameter, we can study the sensitivity of metrics like time-to-hit-data to the caching extent.
- Connectivity level (degree): It represents how tightly connected is the ICN-based WBAN. We use the connectivity matrix, based on our described communication model in Section 3.
- Data Popularity: It indicates how frequent a specific data content is requested. This metric is measured in percentage with respect to other requested data contents. This parameter is represented by a single Poisson process parameter in order to give the content replacements per time unit.
- PNF (%): It is the probability of a physical damage and/or a battery depletion for the deployed WBAN node due to harsh operational conditions. This parameter is chosen to reflect the impact in case of disaster scenarios or fragmented WBAN.

### 5.3. Simulation and Results

The following figures depict the achieved results. Our first objective is to confirm that increasing the extent of caching in ICNs, in terms of both size and number of levels, will reduce time to meet data for all cache policies.

**Figure 2:**
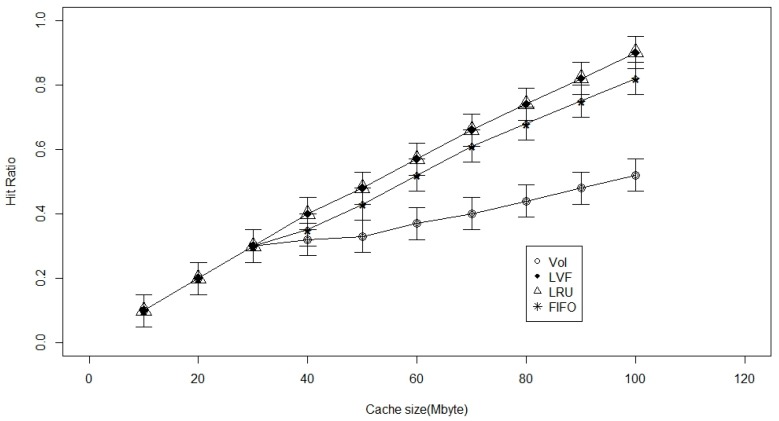
Cache size versus the hit ratio with 1-level caching.

According to Figure 2, we can deduce that the Value of sensed Information is not efficient in a level one cache, however, LVF, FIFO, and LRU have better performance in this case. The performance gains do not increase much under the VoI approach when the cache size is increased beyond 30, due to the limited number of caching levels. Since the decision making is taken only at cognitive nodes (CNs), implementing the VoI-based cache replacement strategy necessitates more resources to achieve better cache hit ratios. This cannot be achieved by only implementing the first level of cache, where the proposed prioritization method will not be effective simply because there are no alternatives to prioritize between them.

**Figure 3:**
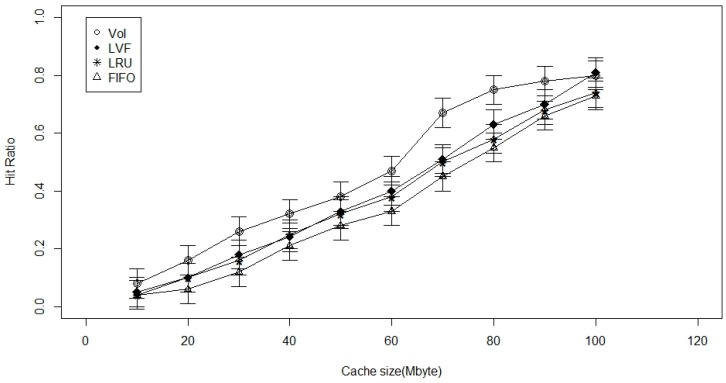
Cache size versus the hit ratio with 2-level caching.

In Figure 3, where we have two levels of caching (one on LCN and the other on RN), we find that the VoI surpasses the other two replacement techniques. The advantage offered here by the VoI-based cache replacement technique is that it can replace data based on the user-requirements. However, in other cache replacement techniques such as LVF, FIFO, and LRU, authors are only worried about the match of the data request packet numbers, irrespective of the age of dat, its popularity, or the delay involved in sensing and transmitting it to the Sink. However, with the VoI-based technique, factors like age of data, popularity, and interference are also considered during the data replacement. Thus, older, easy to get data is replaced by fresher data that are much harder to get. Accordingly, we suggest the use of 2-levels of caches, one at the CN, and one at RN. Since we have only one type of the considered requests, there is a maximum performance gain when the cache size is increased beyond 60 Mbyte. This support our claimed VoI approach against the heterogeneous type of requests in WBAN applications.

**Figure 4:**
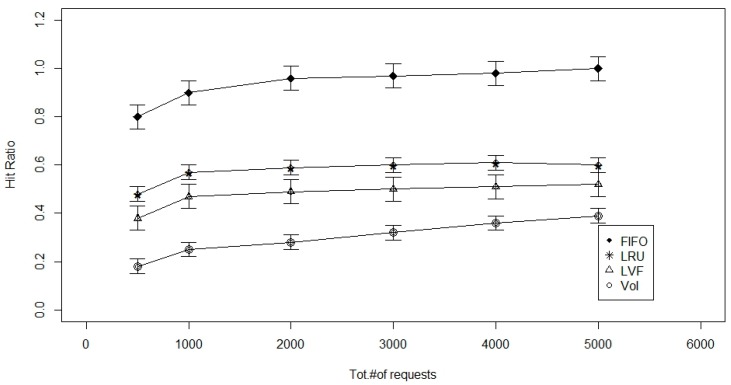
Total number of requests versus the hit ratio with 1-level caching.

**Figure 5:**
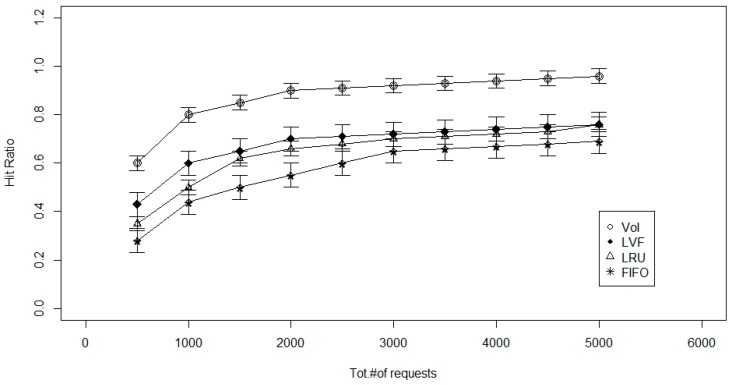
Total number of requests versus the hit ratio with 2-level caching.

The figures for the next set of simulations (Figure 4 and Figure 5) are set to analyze the performance of the cache replacement strategies as the number of requests that a given network needs to serve increases from 500 to 5000 req/h. The cache size is set to be 100 Mbyte and the number of request types are fixed at four.

As seen in the above Figures, the advantage offered by the Value of sensed Information-based cache replacement technique is that it can replace data based on the user-requirements and VoI of data. Other cache replacement techniques are only worried about the match of the data request packet numbers, irrespective of the age of data or its popularity or the delay involved in sensing and transmitting it to the Sink. However, with the VoI-based technique, factors like age of data, popularity, and VoI are also considered during replacement. Older data is replaced by fresher data unlike other techniques that look only for a number match irrespective of its age. Based on this, we suggest the use of 2-levels of caches, one at the LCN, and one at RN. At the LCN, we can use the VoI-based cache replacement strategy, and at RN, we can use either the FIFO or LRU to make the computation less complex. Size of 1st level cache and 2nd level cache is set be 100 Mbyte and number of packet requests is set to be 10,000.

From Table 2, we can see that for the two levels of caches, the best possible combinations are: VoI-based replacement strategy at L1 cache and VoI- or LRU-based replacement strategy at L2 cache. Although FIFO based techniques sometimes perform better in terms of hit ratio, we cannot say that they meet the user requirements in terms of the age of the data and delay requirements. Since the decision making is only at LCNs, implementing the VoI-based cache replacement strategy at LCNs can significantly help the network in saving more resources if a cache hit is found at the first level of cache.

**Figure 6:**
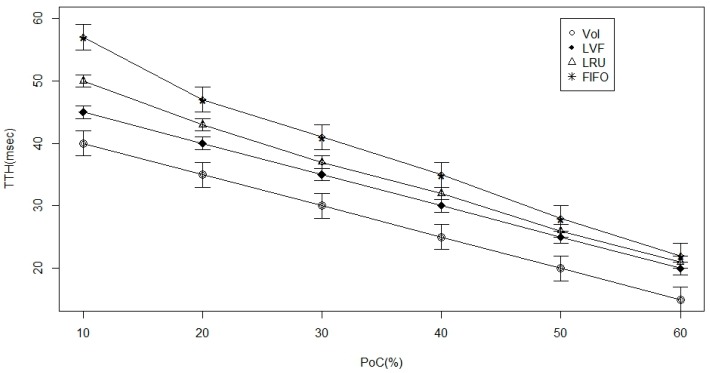
Time-to-hit ratio versus the percentage of nodes with caches in a randomly generated Information Centric Network (ICN)-based Wireless Body Area Network (WBAN).

**Figure 7:**
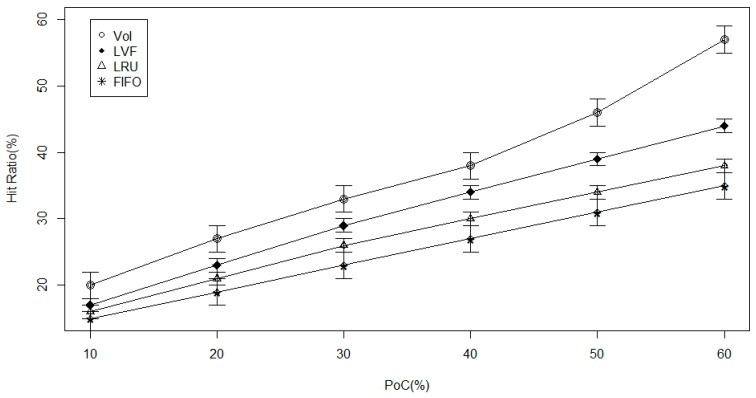
Hit-ratio versus the percentage of nodes with caches in a randomly generated ICN-based WBAN.

Figure 6 and Figure 7, below, represent the findings from the simulation experiment of the 2 level cache. From both figures, the extent of cache availability increases proportionally. According to Figure 6, we observe that the overall time to meet data, which is our main performance metric, is reduced in all performance policies. However, the VoI policy performs best with higher proportions of nodes attached to caches. On the contrary, Figure 7 shows that there is an increase in the data hit for all the approaches, and, hence, we conclude that the VoI is better due to its ability to replace the most relevant data according to an ICN-specific set of attributes.

**Figure 8:**
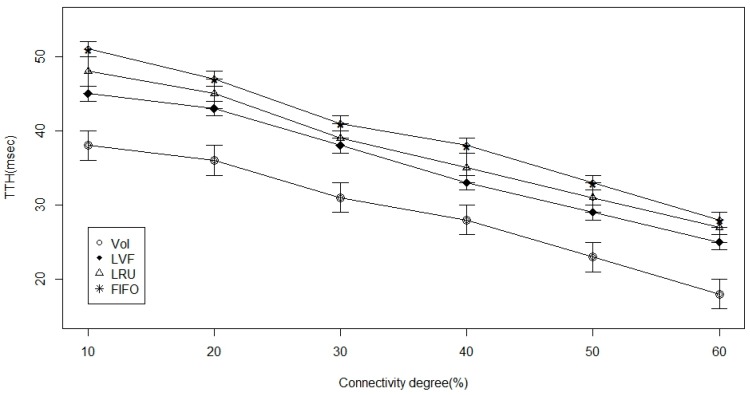
Time-to-hit ratio versus the connectivity degree percentage.

**Figure 9:**
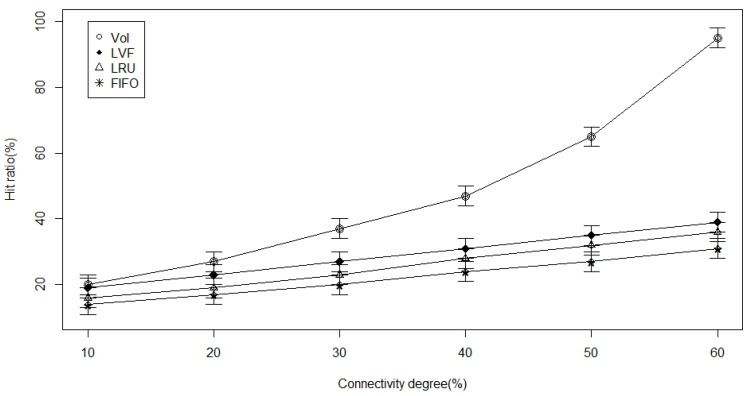
Hit ratio versus the connectivity degree percentage.

**Figure 10:**
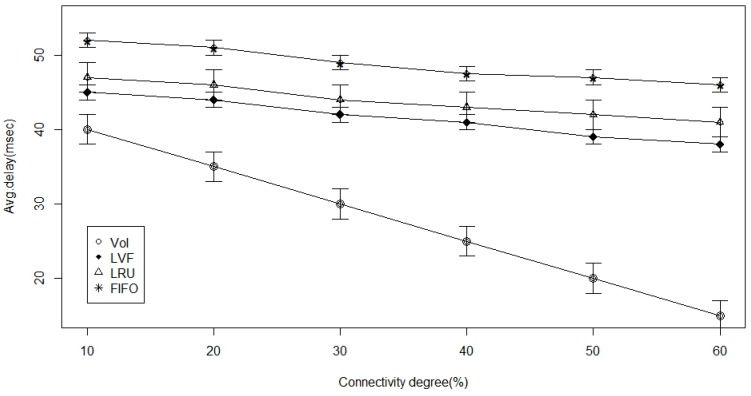
Average in network delay versus the connectivity degree percentage.

**Figure 11:**
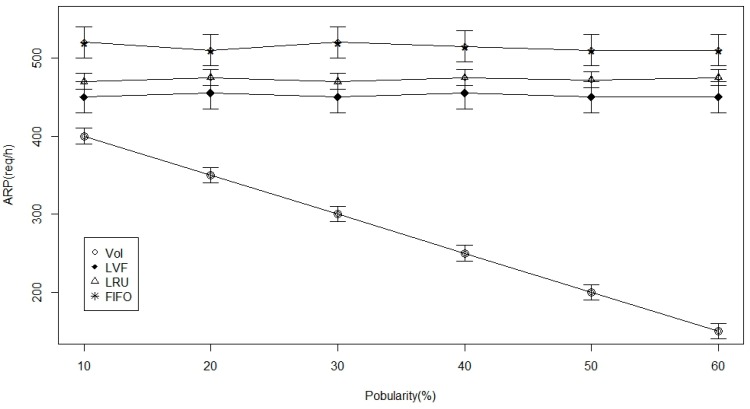
Publisher load versus the data popularity.

In Figure 8, Figure 9 and Figure 10, connectivity level (degree) is the examined parameter. From Figure 8, we deduce that there is an increase in time to hit data as the ICN-based WBAN connectivity increases in all the approaches. However, we notice that VoI is less dependent on the network and, hence, better than the other two approaches. VoI is more dependent on the data type of highly desired property in the ICN-based network. Figure 9 shows the data hit performance against a varying network connectivity degree, and while applying the VoI scheme; we notice that the data hit increases exponentially while the network connectivity increases. Nevertheless, the data hit of the other two approaches increases linearly. Moreover, Figure 10 shows that VoI is the best in terms of delay. This can be attributed to the application of the delay factor while deciding what data to replace. Figure 11 shows the effect of data popularity in terms of publisher load. The VoI tops LRU and FIFO as the popularity metric increases. This is a very desirable property in ICNs.

The VoI-based technique would be suitable for the ICN-based approach, as we propose to use a named data association for the sensed data, such as attribute-value pairs, and the cache size can be decided based on the different types of user requests that the network is expecting to serve. We can expect that the users are more satisfied with the response received from the LCN, as it retains information in its cache based on both data popularity and various parameters that affect gathering sensed information and the energy involved in doing so, as the network scales up to larger sizes.

**Figure 12:**
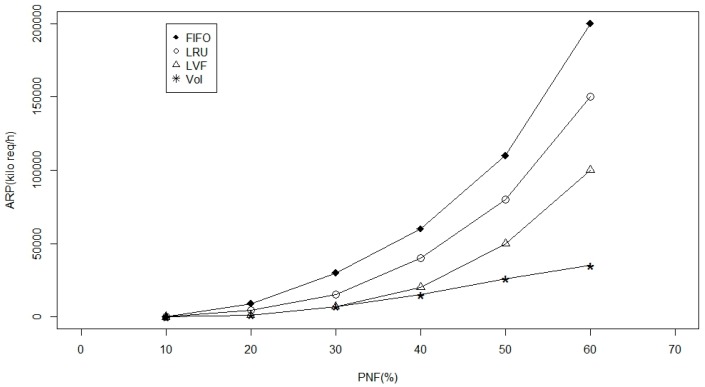
Publisher load versus the probability of node failure in the network.

**Figure 13:**
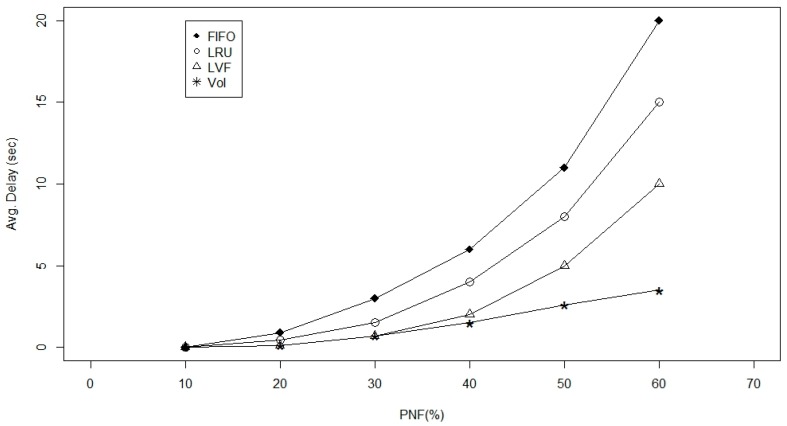
Average delay versus the probability of node failure in the network.

Furthermore, we examined the four caching approaches; LVF, LRU, FIFO and VoI in terms of the average publisher load (Figure 12) and average delay (Figure 13) impacts while considering disaster scenarios and/or fragmented WBAN, where failure of a critical node partitions the network into disjoint segments [30]. Based on Figure 12, we notice a sever effect on the average request per publisher (ARP) while the PNF is increasing. Where all approaches are experiencing an exponential increase in publisher loads as the network becomes disconnected. However, using the proposed VoI approach the increment is going linear, which can be a very desirable feature in WBANs while experiencing harsh operational conditions and sever mobility effects. Moreover, we examined the VoI approach against the other three baselines while varying the PNF values from 10% to 60% to check the effect on the average data delivery delay in Figure 13. Again we can see the exponential grow up in delay while applying the other three approaches against the VoI-based approach. This can be returned to the special care that has been taken while replacing in-network cached data by assigning the highest tuning parameter weight to data popularity factor that helps a lot in disaster scenarios. It is worth mentioning, however, that through the displayed results, LVF approach does always come as a second-best to VoI, with a considerable gap between the two. We explain this as a result of LVF being the closest caching approach to VoI in terms of treating data/information as measurable entities that can be compared and replaced accordingly.

## 6. Conclusions

The VoI-based technique is suitable for use in ICNs which use named data association for the sensed data and are expected to support node mobility in the future. We can expect that the users are more satisfied with the response received from the LCNs, as they retain information in their cache based on both data popularity and various parameters that affect gathering sensed information and the energy involved in doing so, as the network scales up to larger sizes. Further, the VoI cache replacement strategy will help in graceful degradation of the network, as cached data can be provided from LCNs even after sensor node deaths. Moreover, extensive simulations to evaluate the impact of a disjointed (fragmented) network on the average delay and data publisher load have been studied to show the effectiveness of the VoI cache replacement strategy.

## Figures and Tables

**Table 1:** Energy and Quality of Information (QoI) attribute priority for different data traffic types.

	Quality of Information (QoI)—Attributes			
Request Type	Latency (L)	Energy (E)	Reliability (R)	Throughput (T)
Type I: On-Demand	x	3	1	2
Type II: Periodic	1	2	4	3
Type III: Emergency	1	1	x	2

**Table 2:** Two-level caching comparison.

L1 Caching Policy	L1 Hit Ratio	L2 Caching Policy	L2 Hit Ratio	Tot. Hit Ratio
VoI	0.811542	LRU	0.81743	0.81542
VoI	0.547	FIFO	0.7792	0.6194
VoI	0.899	VoI	0.0099	0.81743
LRU	0.754	VoI	0.398	0.6837
LRU	0.9	FIFO	0	0.71818
LRU	0.802	LRU	0.49	0.75125
FIFO	0.9	LRU	0	0.71818
FIFO	0.9	VoI	0	0.61818
FIFO	0.9	FIFO	0	0.65125
